# Usefulness of Three-Dimensional Transthoracic Echocardiographic Planimetry in a 4-Month-Old Infant with Comorbid Aortic Stenosis and Coarctation of the Aorta Complicated with Low Left Ventricular Ejection Fraction

**DOI:** 10.1016/j.case.2022.06.001

**Published:** 2022-07-02

**Authors:** Junpei Kawamura, Kentaro Ueno, Yoshihiro Takahashi, Naohiro Shiokawa, Daisuke Hazeki, Yasuhiro Okamoto

**Affiliations:** Department of Pediatrics, Kagoshima University Graduate School of Medical and Dental Sciences, Kagoshima, Japan

**Keywords:** Three-dimensional echocardiography, Infant, Pediatric, Aortic stenosis, Coarctation

## Abstract

• Comorbid valvular AS, CoA, and LV dysfunction can complicate the order of interventions.
• Continuity equation cannot be used to assess AS severity in the setting of decreased LVEF.
• 3D-TTE planimetry can evaluate AS severity in infants with good acoustic windows.

## Graphical abstract

**Figure:**
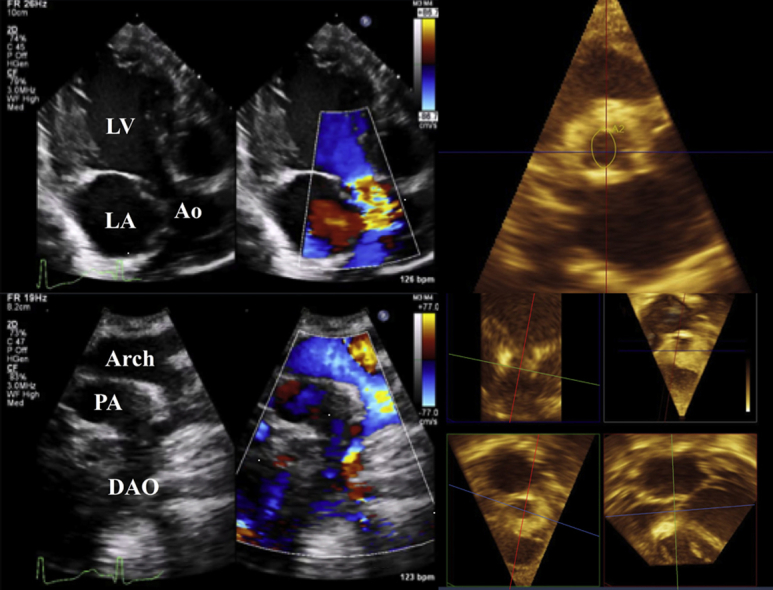

## Introduction

Coarctation of the aorta (CoA) is associated with valvular aortic stenosis (AS) in 50% to 75% of cases, with most cases also involving bicuspid aortic valves.^1^ Surgery of the aortic arch or both the aortic arch and aortic valve is performed in infant patients with CoA and valvular AS on a case-by-case basis.^2^ Cases of comorbid CoA and valvular AS are usually diagnosed within a few days of birth. Surgery for CoA is performed in the neonatal period or infancy, and intravenous prostaglandin E1 is used to maintain the patent ductus arteriosus (PDA) and avoid loss of contractility. However, in infants with comorbid CoA and valvular AS with low left ventricular (LV) ejection fraction (LVEF) caused by afterload mismatch due to spontaneous closure of the PDA, it is difficult to evaluate whether surgery is indicated for valvular AS as a strategy to improve the afterload mismatch, unlike in cases of low-gradient valvular AS with low LVEF or comorbid CoA and valvular AS with normal LVEF.

### Case Presentation

**Figure 1:**
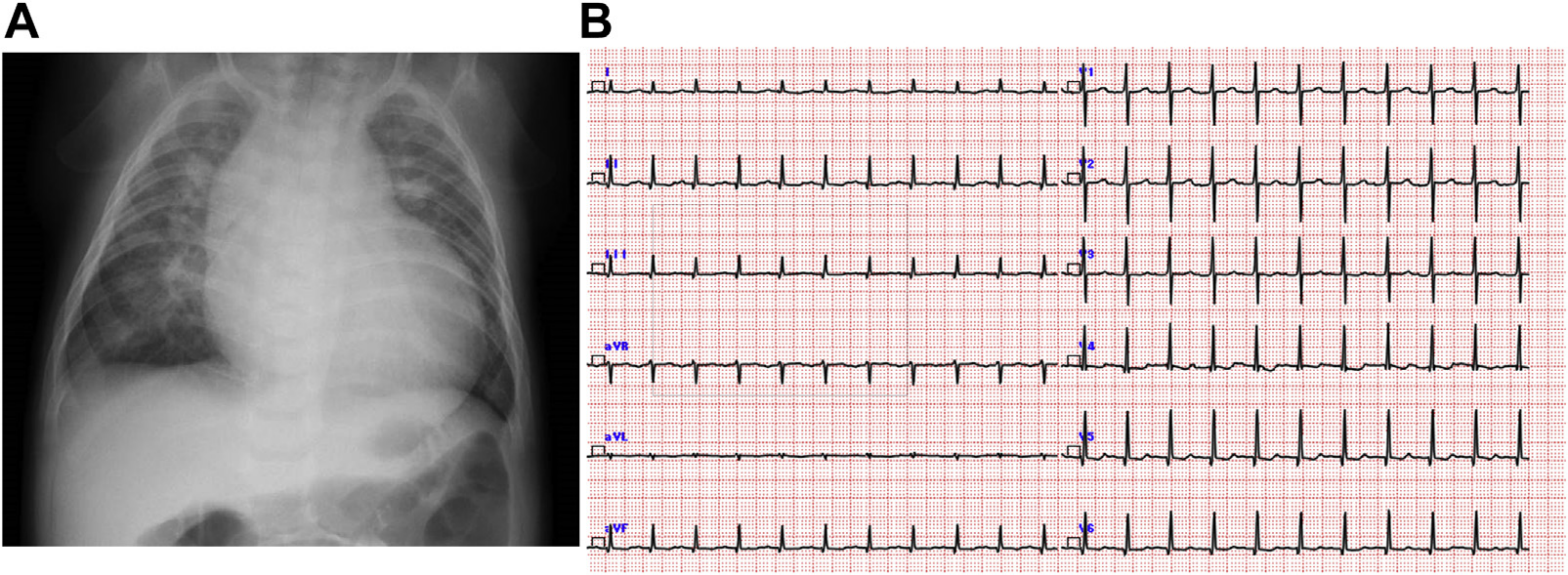
**(A)** Chest radiography revealed a cardiothoracic ratio of 60% with pulmonary congestion. **(B)** A 12-lead electrocardiogram showed sinus rhythm with a heart rate of 133 beats/minute. It also showed LV hypertrophy: positive high-voltage R waves in V5-6 leads, negative high-voltage S waves in V1 leads, and low-voltage T waves.

**Figure 2:**
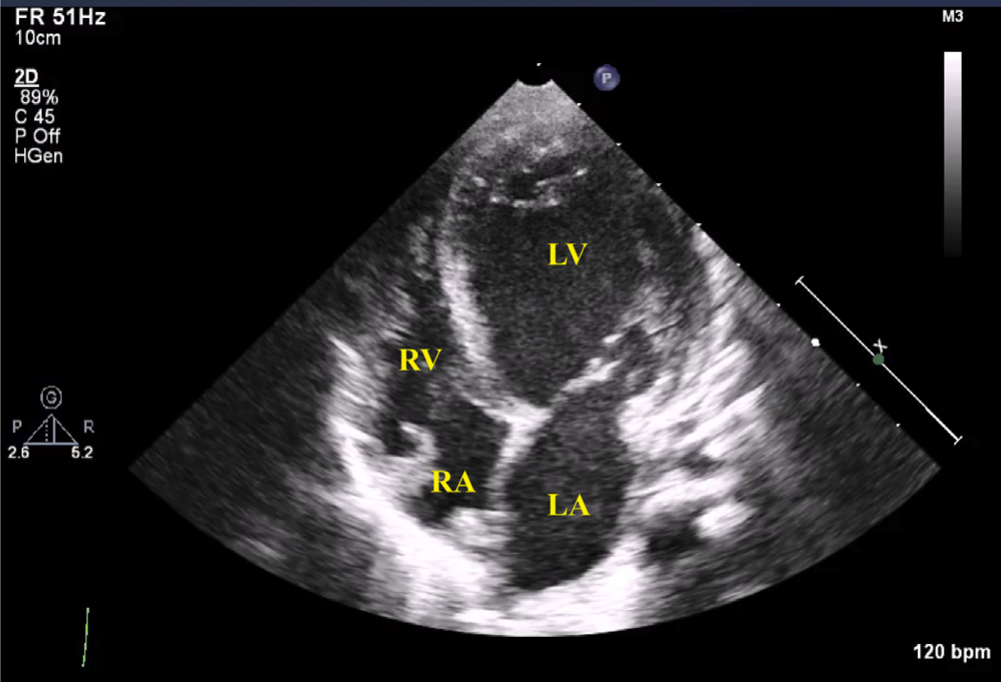
Transthoracic echocardiography from the apical 4-chamber window showing dilated left atrium and left ventricle. *LA*, Left atrium; *LV*, left ventricle; *RA*, right atrium; *RV*, right ventricle.

**Figure 3:**
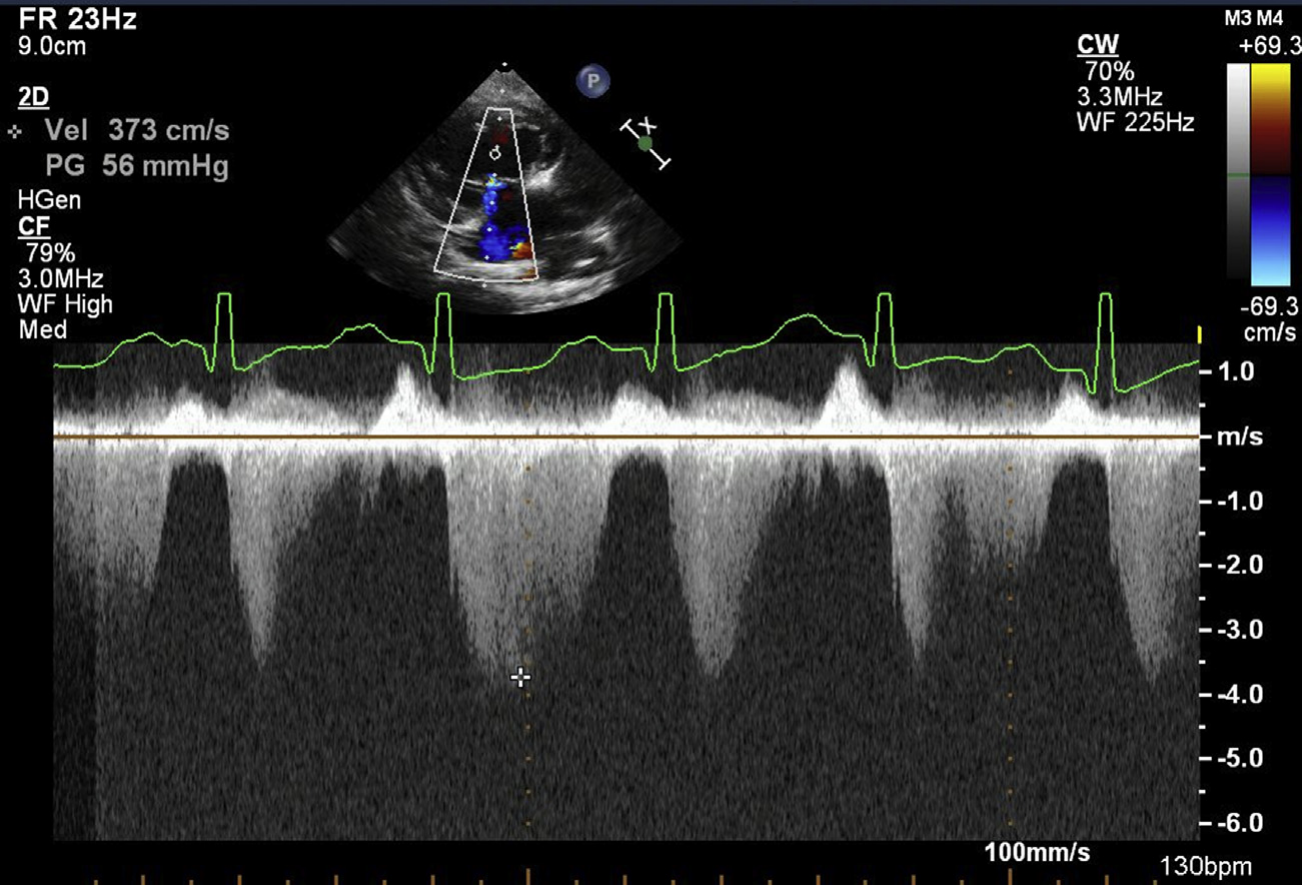
Continuous-wave Doppler assessment of tricuspid valve regurgitation jet from the RV long-axis view of the left parasternal window with RV systolic pressure estimation. The peak velocity was 3.7 m/sec with a calculated peak gradient of 56 mm Hg.

**Figure 4:**
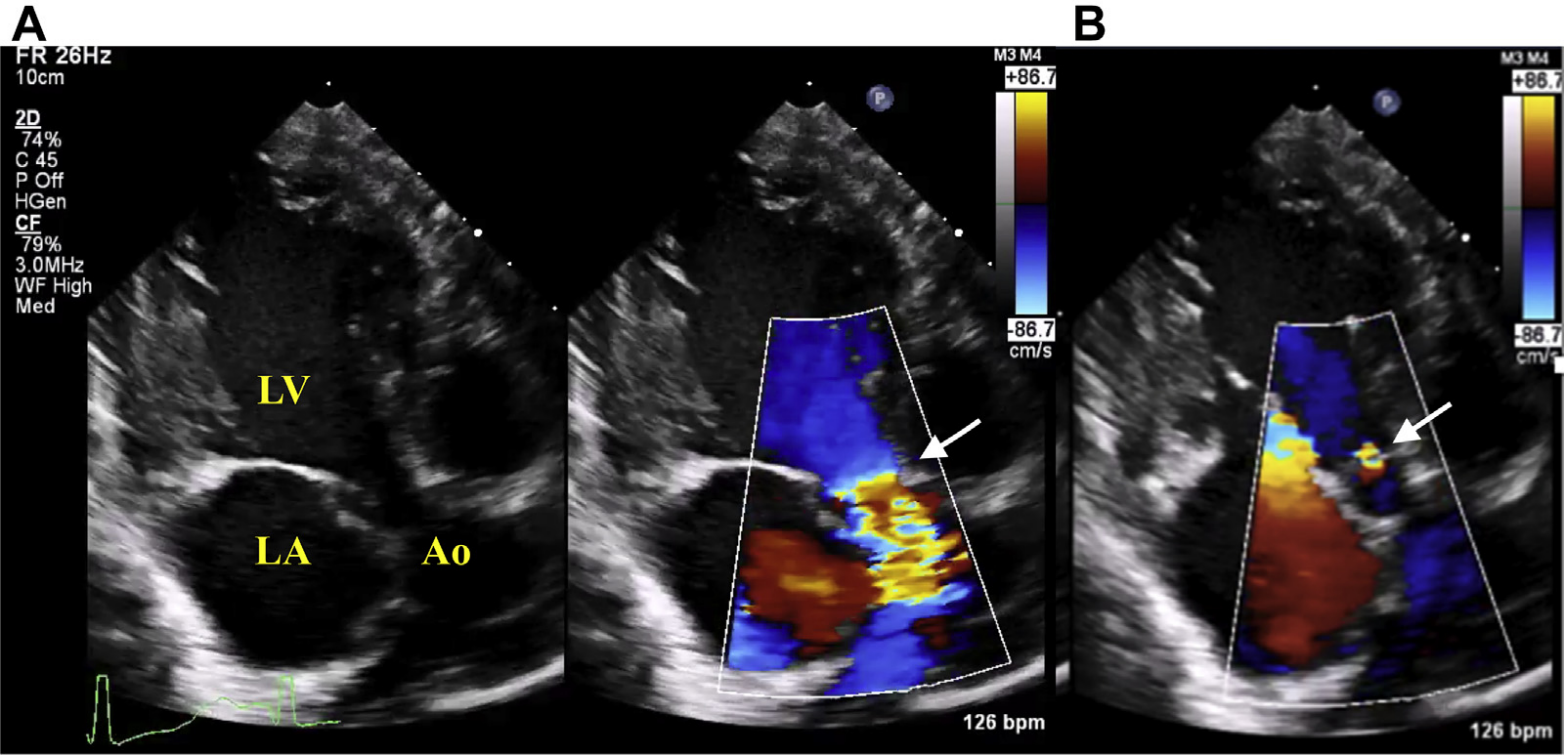
Two-dimensional and color Doppler apical 3-chamber view from TTE showing flow acceleration across the aortic valve in systole (*white arrow*). The peak velocity was 2.7 m/sec with a calculated peak gradient of 29 mm Hg **(A)** and mild central aortic valve regurgitation in diastole (*white arrow*) with vena contracta of 2.0 mm, jet width/LVOT width of 23%, and jet deceleration rate of 516 msec **(B)**. *Ao*, Aorta; *LA*, left atrium; *LV*, left ventricle.

**Figure 5:**
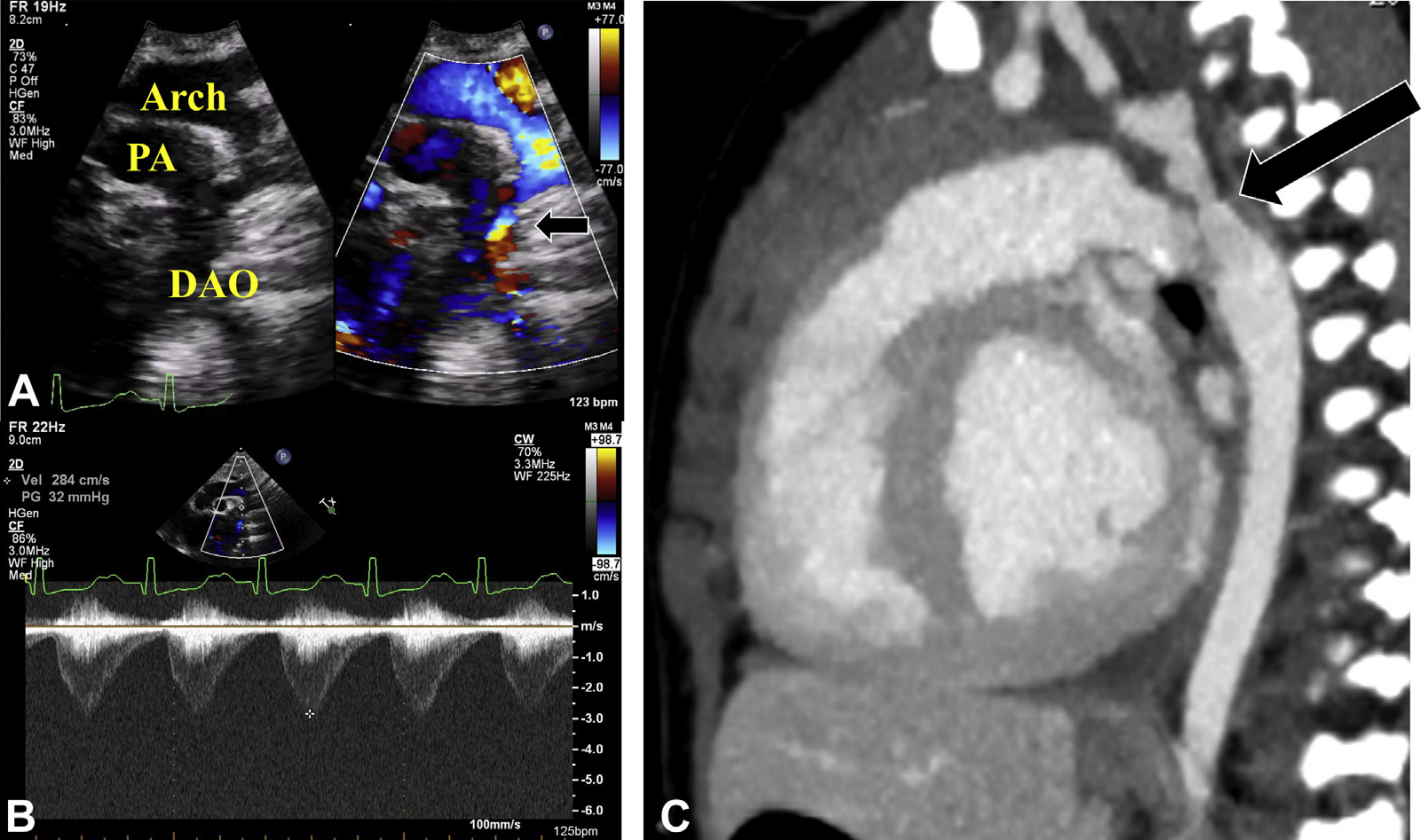
**(A)** Transthoracic long-axis aortic arch view from the suprasternal notch window showing flow acceleration across aortic isthmus (*black arrow*) and **(B)** increased peak velocity (2.8 m/sec with a calculated peak gradient of 32 mm Hg) by continuous-wave Doppler assessment. **(C)** Sagittal view on cardiac computed tomography showing a narrowed aortic isthmus (*black arrow*). *DAO*, Descending aorta; *PA*, pulmonary artery.

**Figure 6:**
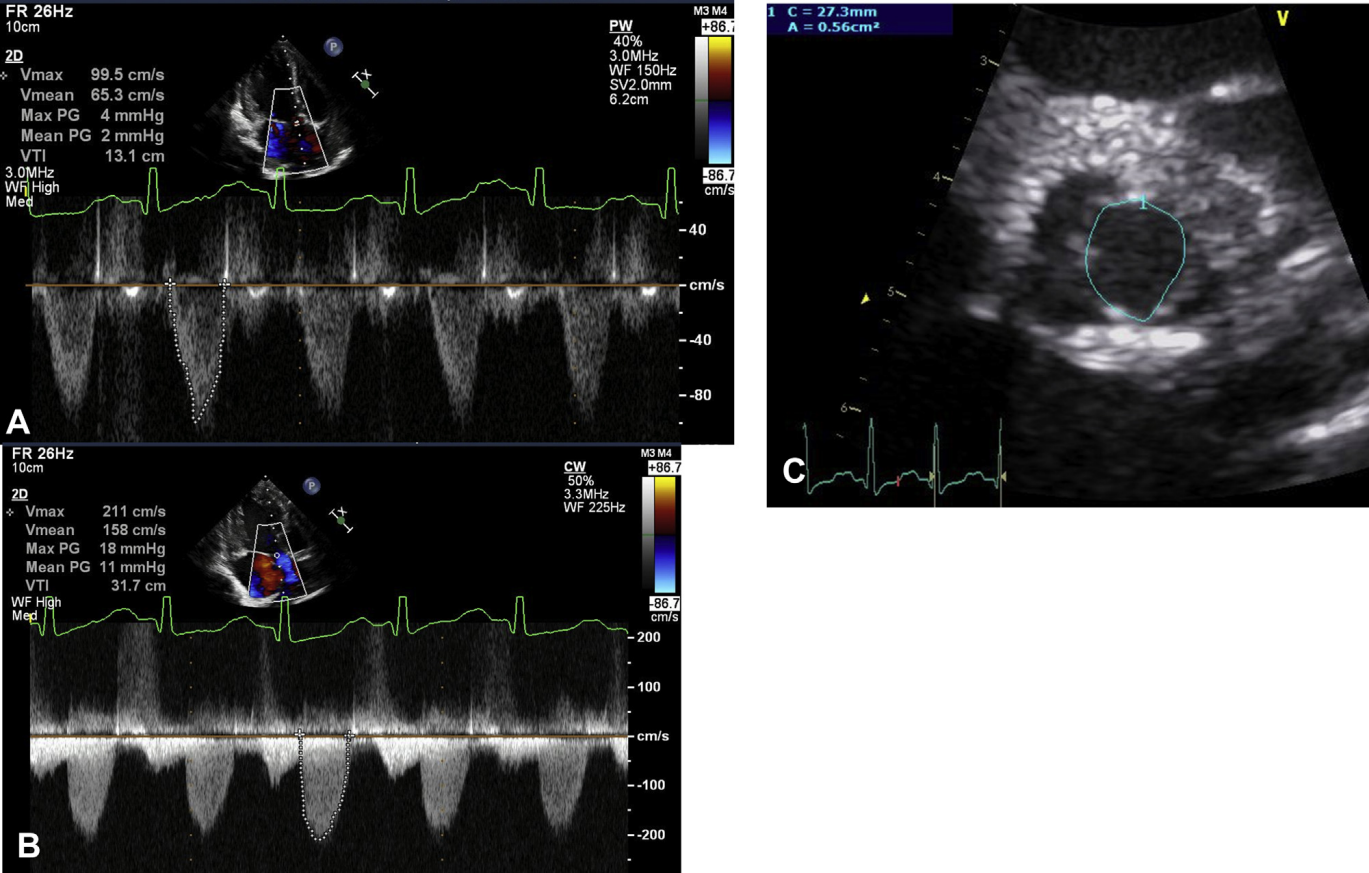
**(A)** Pulsed-wave Doppler echocardiography performed at the level of the LVOT from the apical 3-chamber window. The velocity-time integral was 13 cm. The calculated stroke volume was 7.2 mL. **(B)** Continuous-wave Doppler echocardiography across the LVOT and aortic valve from the apical 3-chamber window. The velocity-time integral was 32 cm. The AVA calculated from the continuity equation was 0.21 cm^2^ (0.62 cm^2^/m^2^). **(C)** Two-dimensional TTE from the left parasternal window shows the aortic valve short-axis view at the midsystolic phase. The traced anatomical AVA calculated from 2D TTE planimetry was 0.56 cm^2^ (1.70 cm^2^/m^2^).

**Figure 7:**
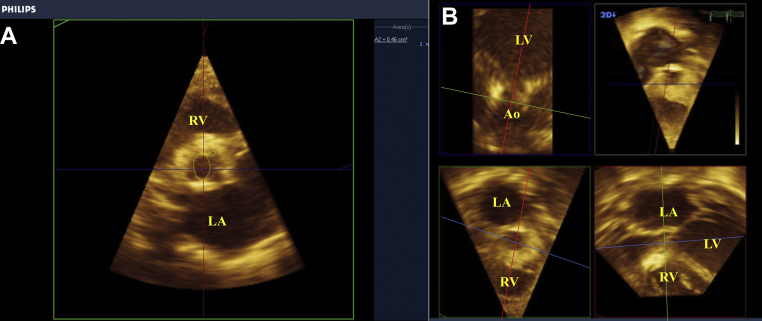
Three-dimensional TTE from the left parasternal window shows the aortic valve short-axis view at the midsystolic phase. **(A)** The 3D-TTE data set was obtained by using 3D analysis software (*top right*). The data set was cropped to create a 2-chamber (*top left*) and a long-axis view (*bottom right*). These made the cross-sectional view at the level of the edges of the aortic valve (*bottom left*). **(B)** The traced anatomical AVA 3D-TTE planimetry in the zoomed cross-sectional view at the level of the edges of the aortic valve was 0.46 cm^2^ (1.39 cm^2^/m^2^). *Ao*, Aorta; *LA*, left atrium; *LV*, left ventricle; *RA*, right atrium; *RV*, right ventricle.

An infant boy born vaginally at 39 weeks' gestation had a history of tachypnea since 2 months of age. Since his feeding and general condition were stable, he was followed at home until 3 months of age. At the 4-month checkup, a heart murmur was noted. The patient presented to a general hospital for a detailed examination and was immediately transferred to our hospital for AS and heart failure. At presentation, the patient had a height of 62.0 cm (25th percentile) and a weight of 6.3 kg (25th percentile) and had tachypnea, was retracting, and had cold extremities. Chest radiography showed cardiomegaly with a cardiothoracic rate of 60% and pulmonary congestion (Figure 1A). Electrocardiography revealed LV hypertrophy and low-voltage T waves (Figure 1B). The brain natriuretic peptide level was high (811.4 pg/mL). Echocardiography showed LV enlargement with an LV end-diastolic diameter of 38.1 mm (*Z* score, +5.2), LV end-diastolic volume of 44.0 mL (133 mL/m^2^), and decreased contractility with a biplane Simpson's LVEF of 31% (Figure 2). The patient had myocardial hypertrophy with an interventricular septum diameter of 8.0 mm (*Z* score, +3.2), LV posterior wall diameter of 5.9 mm (*Z* -score, +4.2), and LV mass corrected by a body mass index of 217 g/m^2^. Moderate mitral valve regurgitation (MR; visible flow convergence, vena contracta of 3.2 mm, mitral inflow of 1.7 m/sec) was noted with tethering of the posterior mitral valve leaflet (Video 1). Secondary pulmonary hypertension was suspected based on elevated right ventricular (RV) systolic pressure estimated from tricuspid regurgitation continuous-wave Doppler gradient of 56 mm Hg and lack of pulmonary stenosis (Figure 3). Echocardiography revealed a bicuspid aortic valve without thickening or calcification, having a raphe between the right coronary cusp and noncoronary cusp (Videos 2 and 3). The annulus was 8.4 mm (*Z* score, +0.2) with accelerated blood flow (peak velocity on continuous-wave Doppler, 2.7 m/sec) with mild central aortic regurgitation (AR; vena contracta of 2.0 mm, jet width/LV outflow tract [LVOT] width of 23%, and jet deceleration rate of 516 msec; Figure 4A and B, Video 4). There was a hypoplastic and long aortic isthmus segment measuring 2.9 mm in diameter (*Z* score, –5.0) with abnormal flow pattern by color and spectral Doppler assessment (peak distal aortic arch uncorrected velocity on continuous-wave Doppler was 2.8 m/sec in the setting of aortic valve stenosis; Figure 5A and B, Video 5). Cardiac computed tomography revealed CoA (proximal arch, 6.9 mm; distal arch, 5.4 mm; isthmus, 2.2 mm; aorta at diaphragm, 5.9 mm; Figure 5C). For the valvular AS, the aortic valve area (AVA) calculated from the continuity equation was 0.21 cm^2^ (0.62 cm^2^/m^2^; Figure 6A and B), and the valvuloarterial impedance was 5.1 mm Hg/mL/m^2^. The stroke volume index was 22.1 mL/m^2^. However, AVA calculated from two-dimensional (2D) transthoracic echocardiographic (TTE) planimetry in the midsystolic phase was 0.56 cm^2^ (1.70 cm^2^/m^2^; Figure 6C), showing a large discrepancy between measures of severity. Preoperatively, it was difficult to decide whether to perform aortic valve surgery in addition to CoA surgery; AVA calculated from three-dimensional (3D)-TTE planimetry (frame rate, 25 fps) in the midsystolic phase was 0.46 cm^2^ (1.39 cm^2^/m^2^; Figure 7, Video 6). Based on the 3D-TTE results, we resolved that the valvular AS was not severe and performed only CoA repair (end-to-side anastomosis under on-pump bypass) 3 days after admission. Postoperatively, there was no re-CoA, and moderate AR (vena contracta of 3.3 mm, jet width/LVOT width of 38%, and jet deceleration rate of 208 msec) was noted. The patient's LV contractility gradually improved but continued to show mildly reduced LVEF (42%). For residual myocardial dysfunction, an oral angiotensin-converting enzyme inhibitor and β-blocker were started. Two years later, the patient's LVEF had improved to 60%, the MR had resolved, and RV systolic pressure estimated from a tricuspid regurgitation continuous-wave Doppler gradient of 10 mm Hg improved; however, mild valvular AS remained, with a peak velocity of 2.5 m/sec (transvalvular pressure gradients, 25 mm Hg). The AVA calculated from the continuity equation was 0.56 cm^2^ (0.93 cm^2^/m^2^), while that calculated on 3D-TTE planimetry (frame rate, 44 fps) at the midsystolic phase was 0.68 cm^2^ (1.13 cm^2^/m^2^). The patient is still being followed clinically for mild valvular AS and mild AR (vena contracta of 2.3 mm, jet width/LVOT width of 19%, and jet deceleration rate of 523 msec).

## Discussion

In this case, indeterminate AS was associated with severe CoA. Since early infancy, low LVEF and left heart failure were observed due to the strong pressure load on the LV caused by the postnatal closure of the ductus arteriosus. Surgical intervention was necessary to reduce the afterload. An estimated 5% to 10% of patients with severe LVOT stenosis have valvular AS and CoA, making it a rare entity.^3^ In this case, 2 causes of LV pressure overload were identified. Although it was clear that the CoA was severe and warranted a surgical repair, it was difficult to determine whether an intervention for the valvular AS was needed. In a previous study, aortic valve balloon valvuloplasty by catheterization was successfully performed in an infant with severe valvular AS and CoA^4^; however, unnecessary treatment should be avoided because a certain percentage of patients who undergo catheterization develop complications such as AR and thrombosis of the peripheral arteries.^4^

In this indeterminate AS associated with severe CoA with impaired systolic function, we evaluated AVA to estimate AS severity (severe is defined as <0.6 cm^2^/m^2^) as the peak velocity on continuous-wave Doppler decreased, contradicting the assessed severity.^5^ In classical low-flow, low-gradient AS (LFLGAS), stress echocardiography can differentiate true severe valvular AS from pseudo severe valvular AS by evaluating unchanged AVA and increased transvalvular pressure gradients after dobutamine administration.^5^ Transesophageal echocardiography (TEE) is useful for morphologically determining true severe valvular AS in a case of unchanging AVA and transvalvular pressure gradients after dobutamine administration or LFLGAS with preserved LVEF (paradoxical LFLGAS).^6^ For these situations, multidetector row cardiac computed tomography is also a useful method for detecting calcification and determining anatomically true AVA.^6^ Low stroke volume index in paradoxical LFLGAS indicates possibly severe valvular AS.^5^ Two-dimensional TTE planimetry is known to overestimate AVA in cases of valvular AS with bicuspid valves.^7^ The continuity equation, a highly accurate evaluation method that can measure functional AVA, is currently the standard evaluation method.^5^ However, in patients with low cardiac function or MR, AVA calculated by the continuity equation may be underestimated.^8^ Thus, we judged the effective orifice area—measured from the continuity equation—in this case as poor despite being suggestive of high severity. Stress echocardiography was contraindicated due to symptomatic heart failure. The 3D planimetry method to evaluate AVA is more accurate than the 2D planimetry method and is useful for supporting surgical decision-making.^9^^,^^10^ Three-dimensional planimetry is more accurate for obtaining the correct cross-sectional view at the level of the edges of the aortic valve than 2D planimetry, because 2D planimetry often makes an oblique cut or cut plane through the valve leaflets. Valvular AS sometimes has (1) thin valve leaflets; (2) significant valve leaflet loss; and (3) calcification, so clear 3D images should be acquired to accurately trace even all aortic valve morphologies. To maintain the accuracy of the 3D measurements, avoiding parallax with 2D estimates/measures from 3D displays to ensure accurate measurements for using the zoom mode and avoiding undergain that overestimates AVA, the accurate angle alignment (the sagittal and coronal planes should be parallel and the transverse plane should be perpendicular to the aortic orifice) and a 50-Hz or higher frame rate to ensure maximal systolic opening (narrowing the imaging range, recording with multiple beats) are necessary. The 3D modality can accurately evaluate the valve leaflet opening as it can measure valves that are heterogeneous, dysplastic, or thickened in pediatric patients.^10^^,^^11^ The 3D-TTE planimetry measurements, in this case, indicated mild valvular AS, unlike the continuity equation. The valvuloarterial impedance is also a useful parameter for LFLGAS, with a value greater than 4.0 to 5.0 indicating severe AS.^12^^,^^13^ The patient had a high valvuloarterial impedance; however, we considered that our patient had decreased contractility and stroke volume due to afterload caused mainly by CoA, which did not directly reflect AS severity. Accordingly, we surgically corrected the CoA, but not the valvular AS, and the contractile dysfunction due to the preoperative afterload improved gradually. Postoperatively, the patient was followed without additional interventions for mild valvular AS. Previous studies have reported that 3D TEE (3D-TEE) is useful for evaluating AVA in valvular AS patients,^14^ but the probe for 3D-TEE could not be used in this infant due to small size,^15^ so we evaluated the use of 3D-TTE. A recent report has assessed AVA calculated from 3D-TTE planimetry, mostly in preschoolers and schoolchildren with only valvular AS,^14^ but we could not find any reports that have evaluated 3D-TTE planimetry in infants with CoA, valvular AS, and reduced LVEF. Three-dimensional TTE has a lower resolution than 3D-TEE; however, 3D-TTE planimetry should be considered in the evaluation of AS severity in infants with concomitant CoA and reduced LVEF. Further comparative examinations are required to confirm 3D-TTE planimetry accuracy in pediatric patients with comorbid CoA and valvular AS.

## Conclusion

Our findings suggest that preoperative 3D-TTE planimetry is a useful technique to be considered when there are discrepant findings on conventional echo parameters, even in cases of low-gradient comorbid CoA and valvular AS with low LVEF and symptomatic severe heart failure.

## Supplementary Data

Video 1 Two-dimensional TTE from the apical 4-chamber window shows the dilated left atrium and ventricle, decreased left ventricle contraction (Simpson's LVEF of 31%), and moderate MR (visible flow convergence, vena contracta of 3.2 mm, mitral inflow of 1.7 m/sec) with tethering of the posterior mitral valve leaflets.

Video 2 Two-dimensional TTE from the left parasternal window shows the aortic valve in the short-axis view. The bicuspid aortic valve has a raphe between the right coronary cusp and noncoronary cusp.

Video 3 Two-dimensional TTE from the left parasternal window shows the aortic valve in the long-axis view. The bicuspid aortic valve was not thickened or calcified.

Video 4 Two-dimensional TTE from the apical 3-chamber window (*left*) with color Doppler (*right*) shows the accelerated flow just from the aortic valve **(A)** and mild aortic valve regurgitation with vena contracta of 2.0 mm and jet width/LVOT width of 23% **(B)**.

Video 5 Two-dimensional TTE from the suprasternal notch window (*left*) with color Doppler (*right*) shows the accelerated flow just from the aortic isthmus with no obvious PDA.

Video 6 Three-dimensional TTE from left parasternal window shows the aortic valve short-axis view. The 3D-TTE data set was obtained by using 3D analysis software (*bottom right*). The data set was cropped to create a 2-chamber (*bottom left*) and a long-axis view (*top right*). The cross-sectional view at the level of the edges of the aortic valve was obtained (*top left*).
